# Integrating Robotics in Hospital and Home Education: A Systematic Review of Innovative Teaching Practices

**DOI:** 10.5334/cie.156

**Published:** 2025-09-01

**Authors:** Federica Pelizzari, Simone Rocco, Simona Ferrari

**Affiliations:** 1 Università Cattolica del Sacro Cuore, IT

**Keywords:** Hospital education, home schooling, K-12, educational robotics, systematic review

## Abstract

This paper presents a systematic literature review (SLR) on integration of robotics in hospitals and home-based educational settings. These schools provide essential educational environments that uphold children’s right to education during prolonged illness. The review explores flexible didactic design, time adaptation, and personalized teaching approaches that are crucial in these contexts. It also examines how digital technologies—specifically coding and educational robotics—enhance pediatric educational experiences, reduce isolation, and improve psychological and social well-being. Coding promotes computational thinking and soft skills, while robotics fosters creativity and leadership, making hospital learning empowering and engaging. A comprehensive search, following the PRISMA framework, was conducted across Scopus, Web of Science, JSTOR, ERIC, and Google Scholar, to identify peer-reviewed studies published between 2010 and 2023. Inclusion criteria targeted studies on robotics in hospital or home education. Grey literature, non-peer-reviewed studies, and research unrelated to non-standard schooling contexts were excluded. Out of 1,500 articles, 30 met the inclusion criteria and were analyzed across four domains: research types and sample profiles, teaching methodologies, technological tools, and skill development. Findings showed that robotics supports educational continuity, fosters engagement, and develops critical skills such as problem-solving, creativity, and leadership. It also reduces social isolation and enhances emotional well-being through interactive, personalized learning. Despite promising results, however, gaps remain, particularly regarding adolescent needs and long-term impacts. This study offers a foundational synthesis for future research and practical applications, emphasizing robotics’ transformative potential in inclusive, future-oriented learning for children in non-conventional settings. It deepens understanding of how robotics addresses educational challenges and provides a base for continued research.

## Theoretical Framework

The right to education is fundamental, ensuring that all students, regardless of their circumstances, have access to quality learning opportunities. For students unable to attend traditional schools due to medical conditions, hospital and home education services play a crucial role in the ability to maintain educational continuity and foste academic, social, and emotional development (World Health Organization, 2022). However, disparities in resources, teacher training, and implementation persist, requiring sustained attention and policy innovation.

According to the Italian Ministry of Education and Merit (Ministero dell’Istruzione e del Merito [MIM], 2023a), during the 2022/2023 school year, 59,226 students benefited from hospital school services, while 2,067 students received home education services, accumulating over 119,198 hours of instruction. Beyond national data, the Eurydice Network (Eurydice Italia, 2023) confirms that hospital schooling is a well-established practice across the European Union (EU), even though the availability of specialized educators, digital tools, and institutional support varies significantly across different regions and countries.

It is therefore urgent to think about innovative teaching practices that can improve continuity, engagement and social inclusion. The integration of digital technologies, adaptive learning environments, and robotics-based interventions offers promising avenues to support these students, not only in terms of academic learning but also in fostering interaction, motivation, and emotional well-being.

Educational technologies have been widely explored as tools to address the challenges of schooling in hospitals and home schooling, but robotics emerges as a unique and particularly promising solution (Chan et al., 2019). *Educational robotics* refers to a class of technologies that combine hardware and software to create interactive systems capable of responding to environmental and social stimuli in an adaptive manner (Gaudiello & Zibetti, 2016). This includes social, assistive, humanoid robots, and programming tools that allow users to build, program, and interact with physical or virtual machines. What distinguishes robotics from other educational technologies is its ability to reintroduce action in a concrete and engaging way, using its tangible and interactive nature to stimulate experiential and multidimensional learning.

Within this context, robotics and digital tools represent a promising avenue for addressing the unique challenges of non-standard educational environments by facilitating interaction, reducing isolation, and enhancing both cognitive and emotional development.

Despite the growing interest in educational robotics, significant gaps remain in literature on the subject. Existing studies tend to focus on specific applications without providing an overview of the potential and challenges associated with these technologies in hospital and home education contexts. A systematic review is needed to synthesize the existing evidence, identify the unique characteristics of several types of robotics, and clarify how these tools can be effectively integrated to meet the needs of this vulnerable population.

This study aimed to fill this gap through a systematic review of the literature on the use of educational robotics in hospital and home settings. The aim was not only to explore the teaching methodologies, sample profiles, and skills developed but also to provide a theoretical and practical basis for future interventions that enhance the potential of robotics in inclusive and personalized education.

### Education in Hospital and Home Schools

Schools in hospital and home schools provide a unique and essential educational environment that ensures the right to education for pediatric patients even during periods of prolonged illness (Kanizsa & Luciano, 2006; Catenazzo, 2019). This context presents specific challenges that require in-depth reflection on the adoption of innovative and effective teaching methodologies capable of responding to the unique needs of pupils in an environment characterized by extraordinary conditions (Lima et al., 2019; Mukherjee et al., 2000). The Italian government’s National Guidelines for Hospital and Home Education underscore the significance of a student-centered approach that is tailored to the individual, necessitating the adoption of flexible teaching methodologies. Such methodologies are designed to ensure that education adapts to the evolving medical and psychological needs of each student who is hospitalised (MIM, 2023b).

Three substantial elements characterize the didactic approach in such contexts (Rivoltella & Modenini, 2012):

**Design:** Design of the didactic action in these contexts cannot be rigid or predefined but must be conceived as a flexible idea adaptable to the changing needs of the hospital context and the specific needs of each pupil (Hargreaves & Fullan, 2012). This type of planning implies a capacity for in-depth observation on the part of the teacher, which becomes a key tool for understanding the peculiarities of each child and adapting educational objectives, work strategies, and activities in a customized manner (Mubin et al., 2013). The ability to observe carefully makes it possible to identify cues for innovative teaching reflections and to calibrate educational objectives according to the individual needs of the children, which can vary depending on their age, experiences, and daily state of health (Catenazzo, 2020).

**Time:** In a hospital and home environment, teaching time does not follow a rigid, predefined schedule as in traditional schools (Shiu, 2004). Instead, time can be extended or contracted according to the child’s clinical situation and their level of involvement in the proposed activities (Lightfoot & Bines, 2000). Such a flexible approach to time optimizes the learning experience, taking advantage of the moments when the child is most motivated or adapting to the needs dictated by medical treatment (Fadlilah et al., 2022; Hsu et al., 2022; Tsao et al., 2017).

**Personalization:** Teachers often work with a single child at a time, adapting their approach to the physical and psychological condition of the pupil as well as their specific educational needs (Thies, 1999). Personalization enables teachers to effectively address the difficulties arising from the disease and to maintain an elevated level of engagement, despite physical and environmental limitations (Konobeev et al., 2020). Each pupil receives individualized attention that considers their uniqueness and contingent circumstances (Lum et al., 2017).

One of the main challenges of teaching in these contexts is the absence of “doing,” which is understood as the action and production typical of traditional school activity (Bobick, 1997). This state of inactivity can negatively affect learning processes (Kucirkova et al., 2019), causing a sense of incompetence in the child and negatively affecting their cognitive abilities (Catenazzo, 2020). To counter these effects, it is essential to reintroduce action at the center of the educational process, promoting activities that involve practical and manipulative dimensions (Mertala, 2021). Creative and expressive activities are particularly well suited to this goal, as they allow different skills to be integrated and concrete products to be produced that highlight the learning that has taken place (Canares et al., 2021; Tsao et al., 2017). Such activities not only meet the needs for flexibility and adaptability required by the day but also help to maintain an important level of motivation and involvement among the children (Wong et al., 2019).

The integration of robotics and digital tools into educational strategies aligns with global policy initiatives such as the Digital Education Action Plan (2021–2027), which advocates for technology-based inclusiveness to ensure equal learning opportunities for vulnerable students (European Commission, 2023). Similarly, UNESCO’s policy briefs on inclusive education highlight the role of technology and robotics in supporting personalized learning experiences (United Nations Educational, Scientific and Cultural Organization, 2021).

### Innovative Potential of Robotics in Education

The role of digital tools in hospital school and home education is crucial, as they offer unique opportunities to enrich the educational experience (Thompson et al., 2015; Wiener et al., 2015). For example, the use of digital tools allows educators to adapt to the diversity of students, facilitating communication and interaction with classmates, reducing feelings of isolation, and improving the psychological and social well-being of hospitalized children (Valls Pou et sl., 2022; Worth, 2002). For children with chronic illnesses, prolonged inactivity not only limits physical movement but also reduces opportunities for meaningful engagement with peers and learning activities (Castro et al., 2021). This lack of action can lead to a deterioration of critical cognitive skills and social competencies, creating a cycle of disengagement and low self-esteem. In such circumstances, providing structured opportunities to reintroduce active participation becomes essential, as it fosters a sense of agency and inclusion that is often compromised by the constraints of illness or treatment (Belpaeme et al., 2018).

The adaptability of educational robotics makes it a valuable tool for personalizing learning experiences in hospital and home education settings. Unlike traditional digital tools, robots can be configured to respond dynamically to each student’s cognitive, emotional, and physical condition. Customizable interaction levels—ranging from simple, directive commands to more complex, conversational engagement—allow educators to tailor robotic interventions based on the learner’s needs and abilities. Additionally, robots can be programmed with content that aligns with individual learning goals, enabling students to progress at their own pace while maintaining engagement and motivation.

Further, for children with limited motor functions, assistive robots can facilitate interaction through alternative input methods such as eye tracking or voice commands, ensuring accessibility and inclusivity. Similarly, for students requiring emotional or psychological support, social robots equipped with adaptive feedback mechanisms can provide encouragement and companionship, helping to reduce stress and enhance resilience. By integrating these personalized features, educational robotics has the potential to transform hospital and home education into a more inclusive, responsive, and engaging learning experience. In hospital and home education settings, where children are often deprived of traditional school routines, the introduction of action—defined as active and productive involvement in educational activities—becomes a fundamental pedagogical priority.

Children are no longer just patients but become active learners, protagonists of their educational journey, able to explore, create, and share knowledge in new and meaningful ways (Giannakoulas & Xinogalos, 2018). Technology, in this context, becomes a bridge connecting the hospital world with the outside world, preserving the sense of belonging to the school community and facilitating reintegration into the traditional educational path once the treatment phase is over.

Specific digital applications for hospital contexts allow children to work on their digital and health skills, offering tools for technological creation and invention that go beyond the simple passive consumption of content (Eguchi, 2017; Rivoltella & Panciroli, 2023). For example, robotics and digital tools can facilitate interaction, reduce isolation, and enhance both cognitive and emotional development in hospital and home education settings (Moerman & Jansens, 2021). Moreover, robot-assisted interventions have been shown to improve emotional support and learning outcomes for hospitalized students (Valentini & Raffaghelli, 2021).

Educational robotics, a type of digital application, is proving to be a powerful tool for hospital education, enabling children to develop emotional, creative, and digital skills (Lee et al., 2019). The effectiveness of robots in educational settings is influenced not only by their functional capabilities but also by their design characteristics, including physical appearance, modes of interaction, and adaptability to specific educational and clinical needs (Slovák & Fitzpatrick, 2015). What makes robotics stand out among other educational technologies is its ability to provide an interactive, tangible experience that reintroduces action in a more direct and meaningful way (Alimisis, 2021). Unlike digital platforms or purely virtual tools, robots allow learners to engage in hands-on activities that combine physical manipulation, immediate feedback, and adaptive interactions (Triantafyllidis et al., 2023). By offering a blend of cognitive, emotional, and social stimulation, robotics—through designing, building, and programming robots—bridges the gap between theoretical learning and practical application, making it particularly suited to non-standard educational contexts such as hospital and home settings (Mubin et al., 2013). This process not only improves their technical skills but also nurtures a sense of competence and autonomy, two fundamental elements for psychological well-being (Kong, 2016; Snow et al., 2019). Social and assistive robots, equipped with emotional and social interaction capabilities, can reduce feelings of isolation and promote emotional involvement (Sharkey, 2016). This is particularly relevant in hospital settings, where children often struggle with a loss of connection with peers and the school community (Rivoltella, 2020).

Educational robotics serves various educational purposes closely tied to their type and the modes of interaction they facilitate. These purposes can be classified as follows (Table 1):

1. *Learning tool:* Robots are frequently employed as tools for teaching technical and cognitive skills. Educational robots, such as LEGO Mindstorms and Bee-Bot, exemplify this role by providing students with hands-on experiences in computational thinking and problem-solving. Through programming interfaces such as Scratch, students interact with these robots in a way that fosters creativity and systematic thinking. The interaction involves direct manipulation of commands and observing the results, making the process interactive and stimulating (Sullivan & Bers, 2018).
2. *Motivator:* Robots like Pepper and NAO are designed to engage students emotionally and socially, enhancing their intrinsic motivation to learn. These social robots utilize conversational capabilities, gestures, and interactive games to make the learning process enjoyable and captivating. For instance, NAO can ask questions, provide feedback, or engage in role-playing scenarios, helping to sustain students’ interest over time (Fletcher, 2023).
3. *Facilitator:* Assistive robots such as PARO are employed to support students with physical or cognitive challenges, enabling them to participate actively in educational activities. These robots facilitate interaction with tools and materials, making education more accessible. The interaction often involves physical assistance, such as grasping objects or aiding mobility, which fosters a sense of autonomy and empowerment (Wada et al., 2010).
4. *Entertainer:* Robots can also function as entertainers, incorporating playful elements into learning activities. For example, robots like Dash and Dot use game-based learning to teach coding and STEM concepts. These robots often rely on interactive games and challenges that keep students engaged and make complex concepts more approachable (Jeong, 2017).

**Table 1:** Classification of Educational Robots Based on Their Purpose, Type, and Mode of Interaction.


*EDUCATIONAL PURPOSE*	TYPE OF ROBOT	MODE OF INTERACTION

*Learning Tool*	Educational robots (e.g., Bee-Bot, LEGO Mindstorms)	Programming, observing results

*Motivator*	Social robots (e.g., NAO, Pepper)	Conversations, interactive games

*Facilitator*	Assistive robots (e.g., PARO)	Physical support, interaction with tools

*Entertainer*	Programmable robots (e.g., Dash, Dot)	Game-based learning, challenges, and games


Robots play a dual role in fostering social and emotional well-being, acting either as mediators of human-human interactions or as direct promoters of emotional bonds. For instance, robots guide group activities and cooperative tasks, enhancing communication and building social connections among participants. On the other hand, robots directly engage users through tactile and auditory feedback, reducing loneliness and promoting emotional resilience. This versatility underscores their potential in supporting both collaborative and individual needs in educational and therapeutic settings.

Educational robotics allows children to express their creativity and develop a sense of competence and leadership in their learning journey (Bers & Horn, 2014; Ferrari & Pelizzari, 2023). This not only enriches educational experience but also provides children with concrete tools to interact with the world around them, improving their ability to cope with everyday challenges and collaborate with others (Dawe et al., 2019).

The integration of educational robotics in hospital education is not merely a methodological choice but represents a pedagogical vision that recognizes the value of education as a tool for empowerment, capable of adapting and responding to complex challenges (Kazakoff & Bers, 2014). This vision embraces the idea that every child, regardless of their health condition, has the right to an education that not only prepares them to face the present but also helps build their future with awareness, confidence, and competence. Inclusive, personalized, and future-oriented education thus becomes the beating heart of the non-standard school—a place where teaching is not limited to imparting knowledge but becomes a process of building identity, resilience, and new possibilities for all children (Taylor, 2022).

Recent advancements in robotics and computational thinking tools, such as Scratch and Blockly, have expanded opportunities for interactive and adaptive learning. These technologies are increasingly recognized for their ability to develop critical skills such as problem-solving, creativity, and collaboration, which are essential in preparing children for the challenges of the 21st century (Belmonte et al., 2023; Zhang et al., 2023).

This innovative teaching approach helps transform the perception of the hospital from a place of care to a place of learning and development (Beran et al., 2015). Despite the growing interest in educational robotics, significant gaps remain in literature on the subject. Thus, the application of these innovations in hospital and home education contexts remains underexplored, and existing studies tend to focus on specific applications without providing an overview of the potential and challenges associated with these technologies in hospital and home education contexts (Cohen et al., 2022). A systematic review is needed to synthesize the existing evidence, identify the unique characteristics of several types of robotics, and clarify how these tools can be effectively integrated to meet the needs of this vulnerable population (Brown et al., 2023). To begin to fill this gap, we conducted a systematic literature review (SLR) that further explored the adoption of robotics in non-standard teaching situations to understand the state of the art in this sector.

## Design and Analysis of the Systematic Literature Review

### Research Questions and Review Type

This research adopted an SLR approach to provide an in-depth and structured understanding of the theories and practices related to integrating robotics in school education for students receiving instruction in hospital or home-based settings. The SLR was chosen for its ability to synthesize existing knowledge, identify emerging trends, research gaps, and future opportunities. This approach allows for a comprehensive review of the available literature, offering critical and rigorous analysis.

For this research, we opted for an “aggregative review,” specifically a realist review (Popay et al., 2006), which is an interpretive method that combines qualitative and quantitative research evidence within specific contexts (Saini & Shlonsky, 2012). When we refer to an “aggregative review combined with an interpretive method,” we are describing an approach that integrates quantitative and qualitative data to provide a more comprehensive understanding of a topic. As such, an aggregative review focuses on collecting and synthesizing findings from multiple studies and organizing them into coherent categories to identify common patterns and relationships. At the same time, the interpretive method adds a level of qualitative analysis that considers context, methodological differences, and aspects that cannot be directly measured, allowing for an understanding of not only “what” emerges from the data but also “why” and “how” this happens. This approach has proven particularly useful for the topic at hand, where the studies analyzed vary widely in terms of methods, samples, and objectives. The integration of the two perspectives allows for a richer and more meaningful synthesis than a mere summation of results.

The synthesis method we selected is the textual narrative synthesis (Littell et al., 2008), an aggregative approach that categorizes studies into more homogeneous groups. This method involves comparing study characteristics, context, quality, and outcomes, highlighting both commonalities and differences (Henderson et al., 2010). As required by the protocol, the SLR contained only primary studies.

The four areas of interest examined in this study were systematically derived from the problem statement and research questions. Given the increasing integration of robotics in education and the specific challenges posed by hospital and home-based instruction, our research aimed to explore how robotics is being implemented in these settings and what impact it has on students’ learning experiences. The research questions were formulated to address the key dimensions necessary for understanding this integration and led to the identification of four principal areas of interest:

1. **Type of research conducted and sample:** This domain explored the types of research (qualitative, quantitative, mixed, etc.) conducted in the context of integrating robotics into hospital and home education and describes the sample involved in such studies (e.g., number of participants, age, specific context). The analysis also included an assessment of the sample size and representativeness in relation to the study’s objectives.
2. **Teaching practices:** This domain focused on the methodologies used to teach robotics. The aim was to analyze which practices have been implemented and adapted to meet the specific needs of hospitalized or domiciled students and to evaluate the effectiveness and efficiency of these approaches.
3. **Tools, platforms, and resources:** This domain identified and analyzed the technological tools, digital platforms, and educational resources used in teaching robotics in non-conventional contexts, assessing their suitability and the characteristics that make them particularly appropriate for such contexts.
4. **Skills developed:** This domain focused on the type of skills—cognitive, social, and technical—developed using robotics. The extent to which these skills contribute to the overall development of students in hospital or home-based educational environments was explored.

The research questions (RQ) that guided the study were as follows:

**RQ1**. What are the most common types of research and sample profiles used in studies on the integration of robotics in hospital and home education?

**RQ2**. What teaching practices are currently used for teaching robotics in non-standard school settings (hospital and home)?

**RQ3**. Which tools, platforms, and resources are used for teaching robotics in hospitals and home settings?

**RQ4**. What skills are developed using robotics in students receiving education in hospital or home?

Based on the above questions, and to ensure the study’s methodological rigor and comprehensiveness, we adopted the PICO framework (Population, Intervention, Comparison, Outcome), a tool used in systematic reviews to structure research questions and outline inclusion and exclusion criteria (Kitchenham, 2012; Uman, 2011), which helped to delineate the parameters of our study. Hence:

- P: The study population included students who receive education in non-conventional settings (i.e., hospital or home-based). These students often have special educational needs due to health conditions that force them to study outside the traditional school context.
- I: The intervention considered was the integration of robotics in the education of these students. This included analyzing the types of research conducted, the teaching practices used, the tools and platforms employed, and the skills developed through these educational technologies.
- C: No direct comparison with a control group or other forms of education was envisaged. Instead, the practices and outcomes documented in the literature were analyzed to understand the trends and effectiveness of different educational methodologies and tools.
- O: The main outcomes of the SLR included mapping the type of research, analyzing teaching practices, identifying the most effective tools and resources, evaluating the skills developed by the students, and identifying the main challenges and opportunities for improving the integration of robotics in hospital and home education.

### Research Protocol and PRISMA System

The selection of studies to be included in the SLR was guided by stringent criteria to ensure the relevance and quality of the evidence collected (Table 2):

**Table 2:** Inclusion and Exclusion Criteria of SLR.


INCLUSION CRITERIA	EXCLUSION CRITERIA

Type of study: Peer-reviewed articles reporting empirical, theoretical, or review studies relevant to the identified research domains were included.	Type of publication: Non-peer-reviewed studies such as unpublished theses, technical reports, opinion articles, or editorials were excluded to maintain a high standard of quality.

Context: Studies had to explicitly address the integration of robotics in hospital or home education contexts.	Inappropriate context: Articles dealing with the teaching of robotics exclusively in conventional school settings without mentioning education in hospitals or at home.

Language: Studies published in languages other than English and Italian unless they contained official translations.	Language: Articles written in English and Italian were included to ensure access to a broad spectrum of international and national literature.

Time range: Studies published between 2010 and 2023 were selected to ensure relevance and up-to-date information.	


A structured search strategy was developed to ensure comprehensive coverage of relevant literature. The search was conducted using databases that contain peer-reviewed sources in the fields of education and technology. The selected databases included:

1. Scopus: A multidisciplinary database including a wide range of peer-reviewed journals.
2. Web of Science: Another highly relevant multidisciplinary database useful for identifying high-impact articles.
3. JSTOR: Especially relevant for studies concerning educational technology and socio-cultural studies.
4. ERIC: Focused on education and, therefore, crucial for identifying studies relevant to teaching in hospital and home settings.
5. Google Scholar: Used to supplement the search with additional articles and for the method of “snowballing” (Wohlin, 2014), which involves reviewing references from key articles to discover additional relevant studies.

The choice of these sources was based on a strategic assessment of their relevance, completeness, and specialization, ensuring interdisciplinary coverage. Specifically, we decided deliberately to avoid exploring social databases, such as Academia and ResearchGate, to prioritize primary and peer-reviewed work and exclude grey literature, guided by the table of reflection proposed by Garousi et al. (2018). The search focused exclusively on open-access articles found in the specified databases. Our collection of articles was managed using Mendeley, a free reference manager for storing, organising, annotating, and sharing references and research data, with each article manually entered by two independent researchers. A list of keywords was defined for the search string in all databases, based on the PICO scheme.

The search string, using Boolean operators (Scells et al., 2020), was as follows:

(computational thinking OR robotic*) AND (hospital* education* OR home* OR non-conventional schooling OR non-standard schooling)

We decided to use asterisks (*) to include in our research all lemmas derived from and related to the object of investigation. To reduce the risk of bias and increase the reliability of the search, study selection and data extraction were conducted by two independent reviewers. Discrepancies between the reviewers were resolved through detailed discussions and, if necessary, the intervention of a third reviewer.

**Figure 1:**
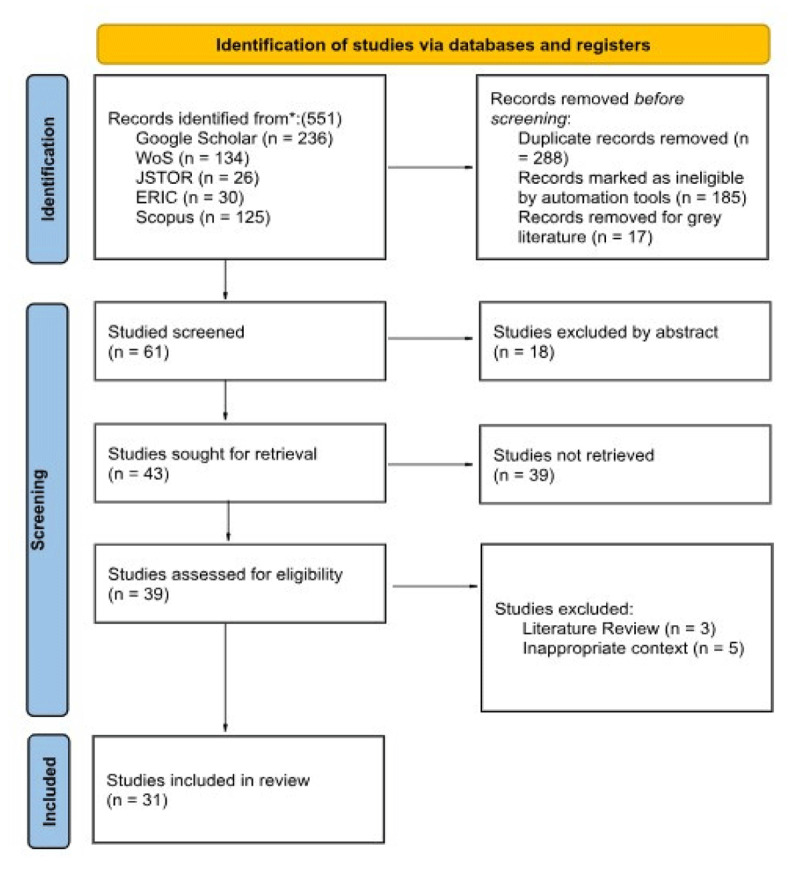
PRISMA Flow of the SLR.

The 2020 version of the PRISMA Flow Chart (Page et al., 2021), an acronym for Preferred Reporting Items for Systematic Reviews and Meta-Analyses, a minimum set of evidence-based items for reporting in systematic reviews and meta-analyses, was used to configure the search. The final search was configured as seen in the PRISMA diagram (Figure 1):

After selecting the articles to be included in the study following the PRISMA framework, a systematic process for data extraction was implemented, designed to ensure the consistency and reliability of the information collected. Duplicates were removed by an automated comparison of title, DOI (digital object identifier), and year of publication, followed by a manual check to identify false positives due to minimal variations in title or metadata. Finally, the extracted data were organized into tables and analyzed qualitatively to identify recurring patterns and trends. The article selection process took place in several stages:

1. *Title and abstract screening:* In this initial phase, the titles and abstracts of the identified articles were reviewed to quickly exclude those that were not relevant to the study. Two independent reviewers participated in this stage, assessing each article to determine its relevance to the inclusion criteria.
2. *Full-text review:* Articles selected during the initial screening were read in full. At this stage, the inclusion and exclusion criteria were again applied to ensure that only the most relevant and high-quality studies were included in the final review.
3. *Resolution of disagreements:* In case of discrepancies between the two reviewers, a discussion was conducted to reach a consensus. If necessary, a third reviewer was consulted to make the final decision.

The accuracy of the dataset was verified through a preliminary exploratory analysis, including cross-checking with key authors and sources to ensure that there were no significant discrepancies.

Following the systematic review process, 30 studies were identified as meeting the inclusion criteria and were subjected to detailed analysis (included in the Supplementary Material 1 and 2). This table serves as a comprehensive reference for the reviewed literature and ensures transparency in the selection and synthesis process.

Publication bias (Higgins et al., 2011), the most significant among others, was mitigated using a complex search string and Boolean operators. For each article included in the review, a standardized data extraction protocol was used, which included the following information:

- Bibliographic information: Authors, year of publication, title, journal of publication.
- Objectives and research questions: Clear description of the objectives of the study and the research questions asked.
- Methods and types of study and sample: Description of the research methods, the type of study applied, and the sample to which the study referred.
- Teaching practices: Description of the teaching methodologies used for teaching robotics, with particular attention to practices adapted for students in unconventional contexts.
- Tools and resources: Information on the technological tools, platforms, and educational resources used, including their characteristics and effectiveness.
- Skills developed: Analysis of the cognitive, social, and technical skills developed using robotics.

Thematic analysis was used to identify recurring patterns, divergences, and gaps in literature.

Two biases could not be fully addressed: *linguistic restriction*, which, by including only studies in English and Italian, may have excluded important research published in other languages; and *methodological heterogeneity*, as the included studies may differ significantly in their methodological approaches, making comparability and synthesis of results difficult.

The key findings of this systematic review are summarized in Appendix 2, which categorizes the types of robots, educational purposes, modes of interaction, and outcomes identified across the analyzed studies. This table serves as a synthesis of the diverse applications and implications of robotics in hospital and home education.

## Results

**Figure 2:**
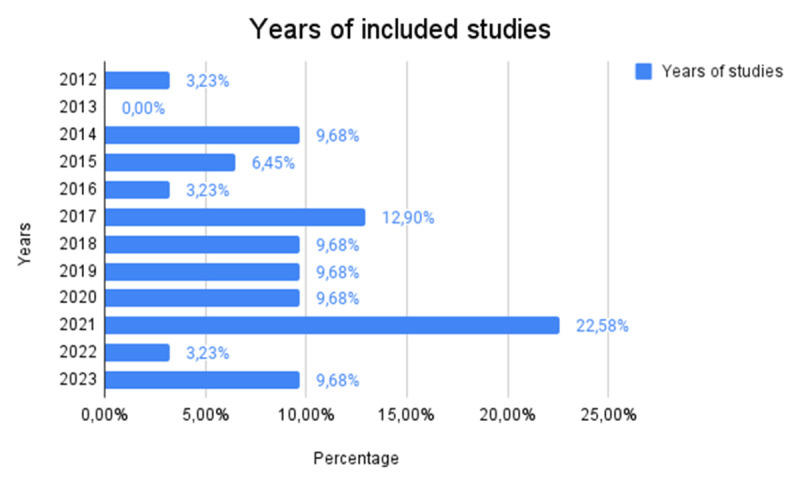
Percentage Distribution of Studies Published per Year.

The Figure 2 shows the percentage distribution of the number of studies published in different years, providing an overview of the growth of research over time. The peak observed in 2021, when there was a significant increase in the number of published studies (22.58%, *n* = 7), can be attributed to several concomitant factors. The COVID-19 pandemic prompted many schools and educational settings to innovate rapidly, adopting advanced educational technologies to ensure continuity of learning. This scenario has fostered an increased interest in educational robotics, both for its potential to support students in non-traditional contexts and for its role in reducing social isolation and promoting well-being. In addition, 2021 saw a rapid evolution of robotic technologies and an increase in funding for research projects in the field of technology education, especially in hospital and home settings. This combination of factors has contributed to an increase in studies exploring the innovative use of robotics in education.

Between 2014 and 2020, the distribution of studies was stable, with several years showing a similar percentage (9.68%, *n* = 4), including 2014.

**Figure 3:**
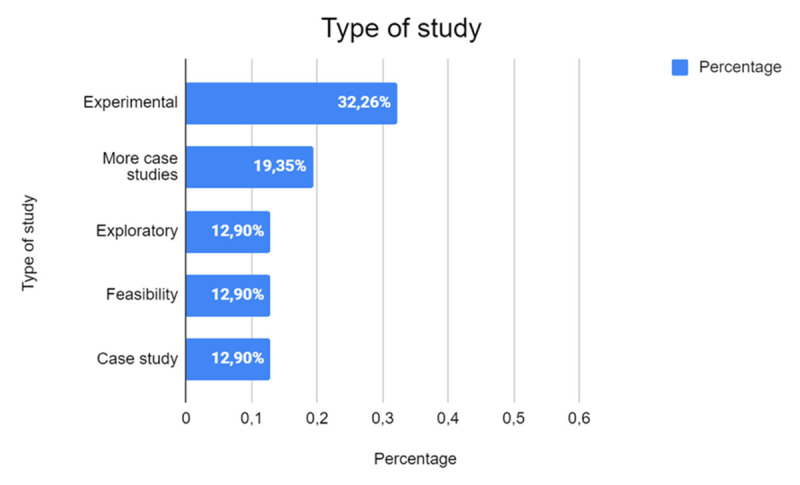
Distribution of Study Types in the SLR.

The first point of interest in the analysis pertains to the research methodologies employed, which show a diverse approach to studying human-robot interactions in educational contexts, as seen in Figure 3. That is, approximately one third of the studies adopt an experimental methodology (32.26%, *n* = 10), highlighting a growing emphasis on practical, evidence-based approaches to assess the effectiveness of human-robot interactions. Experimental studies are particularly valuable in establishing cause-and-effect relationships, enabling researchers to draw conclusions about how robots influence learning outcomes under controlled conditions.

In addition, the use of case studies (32.26%, *n* = 10) and exploratory or feasibility studies, though slightly less common, underscores a complementary focus on understanding specific situations through detailed observation and contextual analysis. Single-case studies delve deeply into implementations, such as a specific classroom, robot type, or demographic group, offering nuanced insights into the interplay between technology and educational processes. In contrast, multiple-case studies extend this approach by comparing results across various contexts, thereby offering a broader perspective on the applicability and scalability of robotic interventions in diverse educational settings.

**Figure 4:**
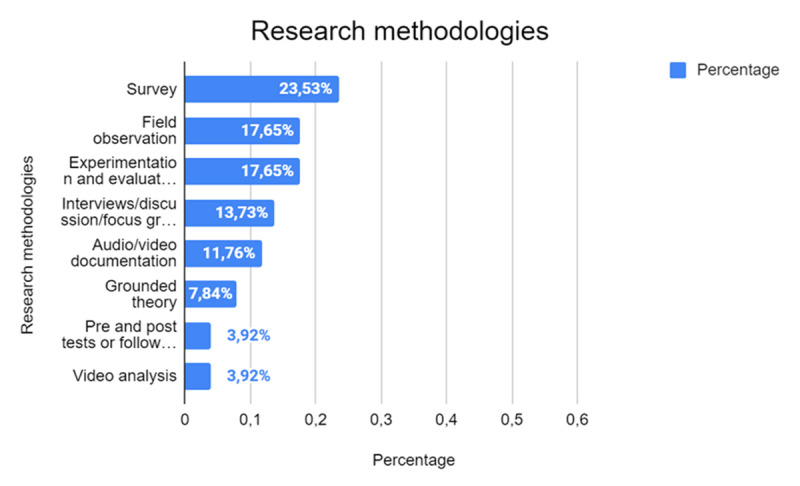
Overview of Research Methodologies Employed in the Analyzed Studies.

The research methods employed in the studies (Figure 4) included in this SLR reveal a wide array of approaches aimed at exploring human-robot interactions in educational contexts. Surveys emerge as the most common method (23.53%, *n* = 7), providing a quantitative lens through which to assess participants’ perceptions, experiences, or attitudes toward the use of robots in learning environments. Surveys allow for the collection of data from a broad population, facilitating generalizability and trend analysis.

Field observations (17.65%, *n* = 5) and experimentation and evaluation (17.65%, *n* = 5) are also prominently used. Field observations enable researchers to gather qualitative insights by directly observing interactions and behaviours in real-world educational settings.

Other methodologies, while less frequently used, contribute to a richer understanding of the field. Interviews, discussions, and focus groups (13.73%, *n* = 4) provide an avenue for in-depth exploration of participants’ experiences, challenges, and reflections, offering valuable qualitative data. Audio/video documentation (11.76%, *n* = 3) supports the detailed analysis of interactions, behaviours, and processes, often serving as a foundation for further qualitative or mixed-methods analysis.

Grounded theory (7.84%, *n* = 2) is another noteworthy methodology, emphasizing the generation of theories directly from data. This approach allows for the emergence of new concepts and frameworks that are deeply rooted in observed phenomena. Pre- and posttests or follow-ups, as well as video analysis (3.92%, *n* = 1 each), though used sparingly, provide critical insights into learning progression, behavioural changes, and interaction patterns over time.

**Figure 5:**
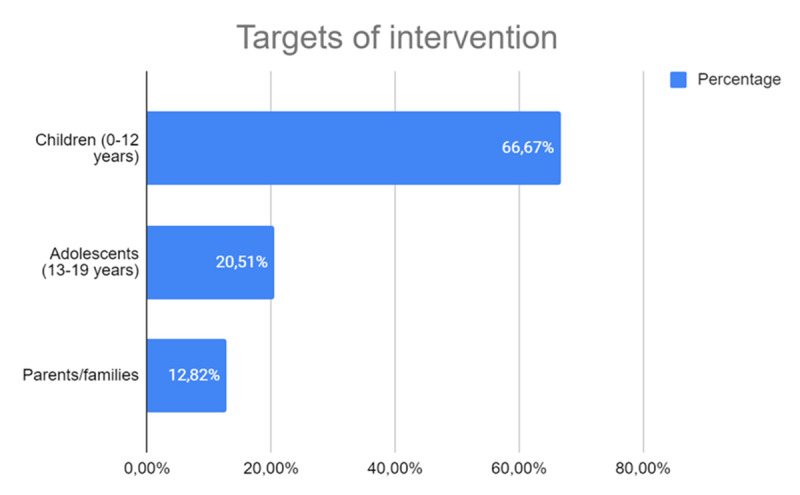
Age Groups Targeted in Studies Using Robotics in Educational Contexts.

In terms of intervention targets (Figure 5), many studies emphasize children aged 0–12 years (66.67%, *n* = 20), reflecting a significant scientific interest in exploring robotic interactions within this critical developmental stage. This focus aligns with the recognition of childhood as a formative period, where exposure to innovative technologies, such as robots, has the potential to shape cognitive, social, and emotional development.

In contrast, the smaller number of studies addressing other groups—such as adolescents, adults, the elderly, and parents—reveals a pronounced disparity in research focus. These groups account for a much lower proportion of the literature, suggesting an underexplored area with untapped potential.

**Figure 6:**
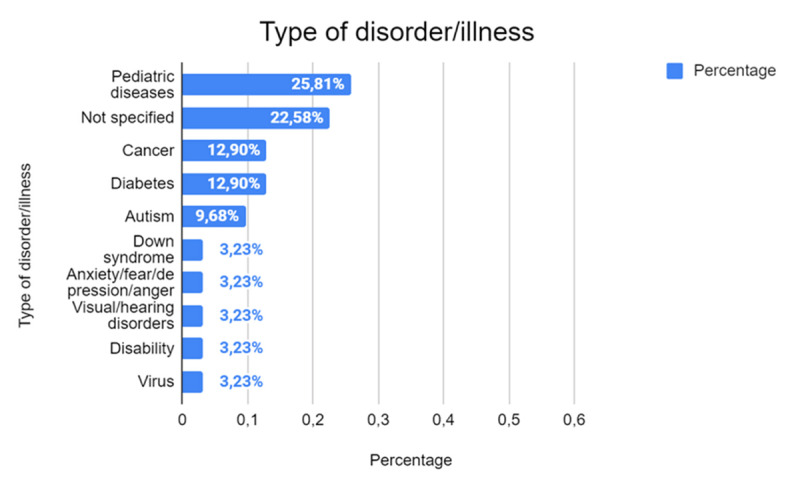
Conditions and Disorders Addressed in Studies on Robotics.

Regarding the types of diseases or disorders supported by using robots (Figure 6), 25.81% (*n* = 8) of the studies focus on pediatric conditions, reflecting the predominant interest in the application of robotics to improve the educational experience and well-being of children. In a significant percentage of cases (22.58%, *n* = 7), the specific disease or disorder is not made explicit, suggesting that these studies are exploratory or aim to evaluate the general effectiveness of robots in non-standard educational contexts. Specific conditions, such as cancer and diabetes, are addressed in 12.90% of the studies (*n* = 4), while autism is represented in 9.68% of the cases (*n* = 3). This highlights a particular interest in areas where robotics can play a significant role in enhancing cognitive, social, and emotional engagement. Other conditions, such as Down syndrome, anxiety, depression, and sensory disabilities, are less frequently supported (3.23%, *n* = 1), indicating that these areas of application are still in their infancy or less explored.

**Figure 7:**
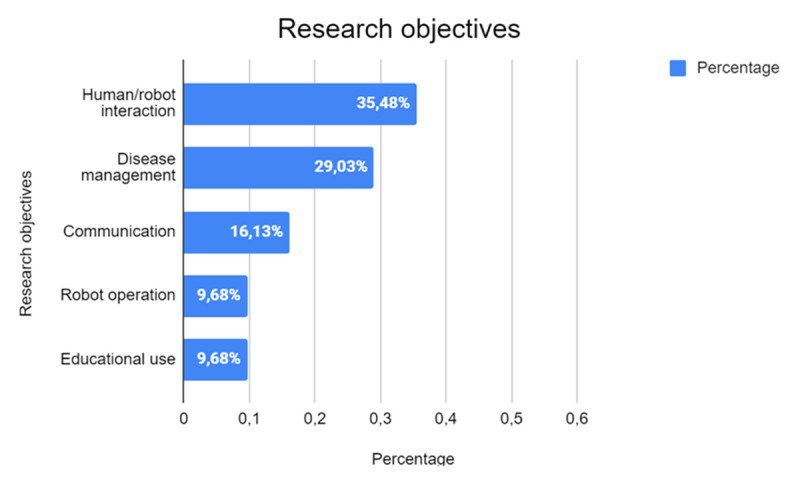
Main Research Objectives in the Analyzed Studies.

The analysis of the data emerging from the studies highlights that the predominant research category is human-robot interaction (Figure 7), featured in half of the studies (35,48%, *n* = 13). This considerable proportion underscores the scientific community’s strong emphasis on understanding and refining the dynamics between humans and robots. The sustained interest in this category reflects its broad potential for application across various fields, including social, educational, and therapeutic contexts. Research in this area seeks to unravel the complexities of interaction to enhance user experience, optimize robotic design, and maximize the effectiveness of robots in addressing human needs.

**Figure 8:**
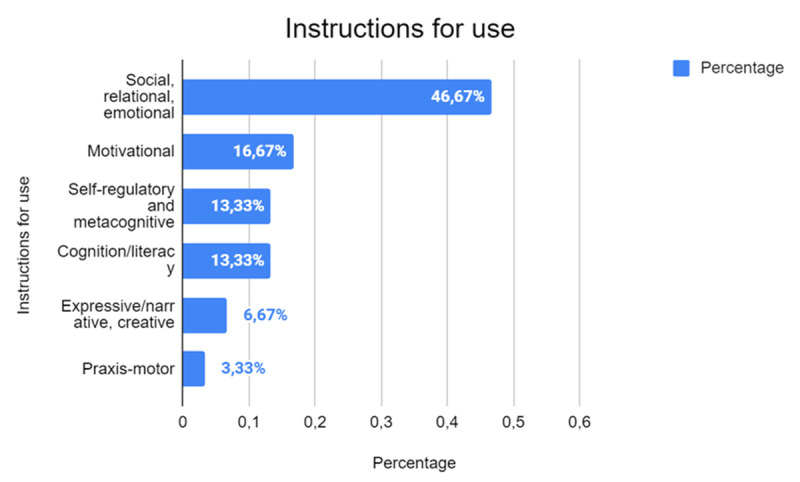
Types of Instructions and Guidance Provided for Using Robots.

The ways in which robots are utilized in the studies analyzed (Figure 8) reveal a predominant focus on social, relational, and emotional aspects, which are the central theme in half of the research (46.67%, *n* = 14). This trend underscores the growing perception of robots as tools capable of enhancing quality of life through positive and meaningful interactions. Such applications are particularly critical in environments like hospitals or long-term care facilities, where emotional support provided by robots can significantly impact patients’ well-being, alleviating stress and fostering a sense of companionship.

Beyond their role in emotional and social support, robots are also explored for their potential in fostering motivation (16.67%, *n* = 5) and promoting autonomic regulation and metacognition (13.33%, *n* = 4). These findings indicate that robotics extends beyond its utility in social contexts to serve as an innovative tool for educational and motivational purposes. Studies focusing on motivation highlight how robots can engage individuals in learning or therapeutic activities, encouraging persistence and active participation. Meanwhile, research into autonomic regulation and metacognition suggests the potential of robots to aid in self-regulation and reflective thinking, offering users opportunities to develop critical skills for personal and academic growth.

**Figure 9:**
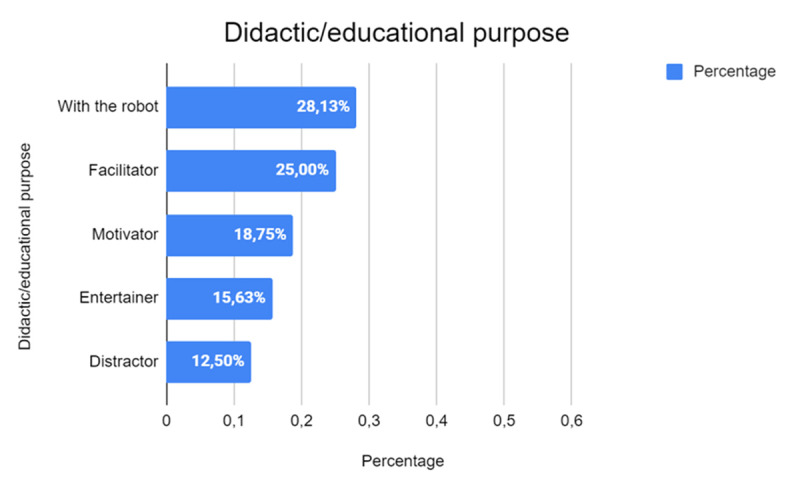
Educational Roles Attributed to Robots in the Reviewed Studies.

The Figure 9 highlights the different educational purposes associated with the use of robots, clearly showing that the predominant role attributed to robots in the literature is as a learning tool, represented by 28.13% (*n* = 9) of the studies. This finding suggests that one of the main purposes of integrating robots into educational settings is learning how to collaborate directly with the robot. This approach not only brings students closer to robotic technology but also prepares them to interact with complex systems, developing technical and operational skills that are increasingly relevant in the contemporary world.

The second most common educational purpose is that of the robot as a facilitator, indicated by 25.00% (*n* = 8) of the studies. This role highlights the importance of robots as a learning support, where technology acts as a means of making the educational process more accessible and manageable for students. In this context, the robot is not just an object of study but becomes an active player in the teaching process, facilitating understanding and knowledge acquisition in a more interactive and engaging way.

Other significant robot roles include that of motivator, represented by 18.75% (*n* = 6) of the studies, and entertainer, represented by 15.63% (*n* = 5). The motivator role is particularly interesting because it suggests that robots can be used to stimulate student interest and engagement, making learning not only more effective but also more challenging. This can be particularly useful in educational settings where student motivation is often a challenge. The entertainment aspect, on the other hand, highlights how robots can make the learning process more enjoyable and less burdensome by incorporating playful elements that can help keep students’ attention and interest high.

Finally, the distraction role, which accounts for 12.50% (*n* = 4) of the studies, indicates that in some contexts the robot is used to divert attention from stressful or tricky situations, as might be the case in therapeutic settings or in challenging learning situations. This role, although less prominent than the others, suggests that robots can also be used strategically to manage the learning environment and improve the overall student experience.

**Figure 10:**
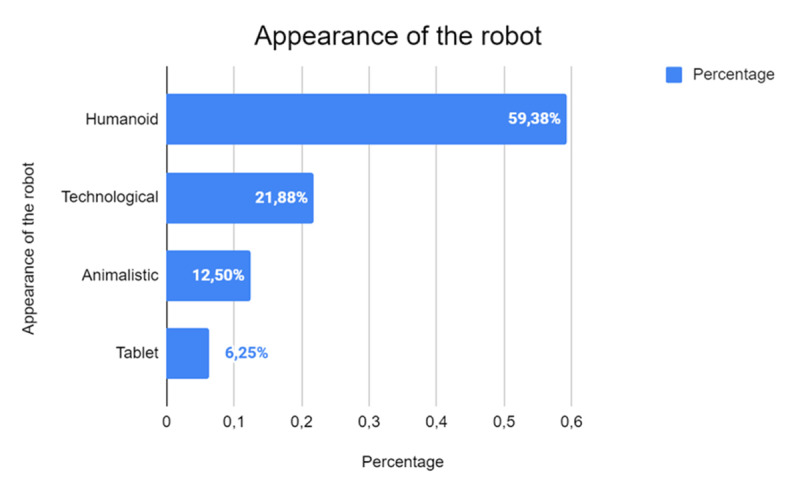
Distribution of Robot Designs Used in the Analyzed Studies.

Analyzing the distribution of robot types used in the studies (Figure 10) reveals a predominant preference for robots with a humanoid appearance, which account for 59.38% of the total (*n* = 19). This finding is particularly significant, as it indicates that humanoid designs are widely perceived as more acceptable and effective in facilitating social interactions. The human-like features of these robots enhance their ability to foster empathy, establish rapport, and communicate effectively, making them especially valuable in contexts such as education and therapy, where interpersonal dynamics are central.

The second most common category is robots with a technological appearance (21.88%, *n* = 7), characterized by designs that emphasize their mechanical or digital nature rather than human resemblance. These robots may be particularly suited to tasks requiring clear differentiation between human and robotic roles, such as in STEM (science, technology, engineering, math) education.

Robots with an animal appearance represent a smaller but still notable portion of the studies (12.50%, *n* = 4). Their design is often intended to evoke feelings of comfort and approachability, leveraging the familiarity and emotional resonance of animals to create engaging and non-threatening interactions.

The choice of robot appearance is closely tied to the specific objectives of the interaction and its contextual requirements. This distribution highlights the importance of aligning robot design with the intended purpose to maximize their effectiveness and acceptance in various domains.

**Figure 11:**
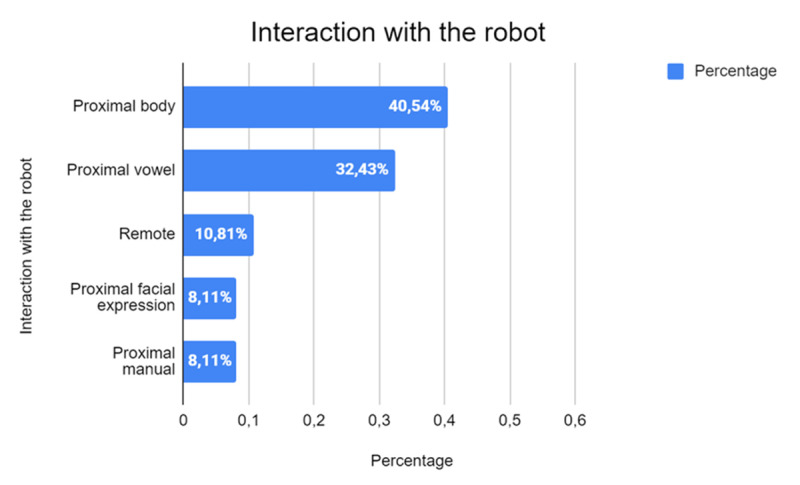
Modes of Interaction with Robots.

Figure 11 shows the different modes of interaction with robots studied, highlighting that many studies focus on Proximal body-type interactions, which constitute 40.54% of the total (*n* = 13). This predominance underscores the importance placed on physical and close interaction, which is considered crucial in improving the effectiveness of the support offered by robots in various application contexts.

The second most common type of interaction, proximal verbal, accounts for 32.43% of the studies (*n* = 10) and is also based on physical proximity but emphasizes verbal communication between the robot and the user. Less common are remote interactions, which account for 10.81% of the studies (*n* = 3). This type of interaction occurs without direct physical contact and often involves the use of telepresence or remote-control technologies. Although remote interaction offers advantages in terms of safety and accessibility, it may not provide the same level of personal and emotional involvement as more physical and close interactions.

Finally, interactions based on facial expressions (proximal facial expression) and hand gestures (proximal manual) are the least represented, with both categories at 8.11% (*n* = 2 each). These forms of interaction, although important for enriching the interaction experience, seem to be used in a complementary way rather than as the focus of the studies.

**Figure 12:**
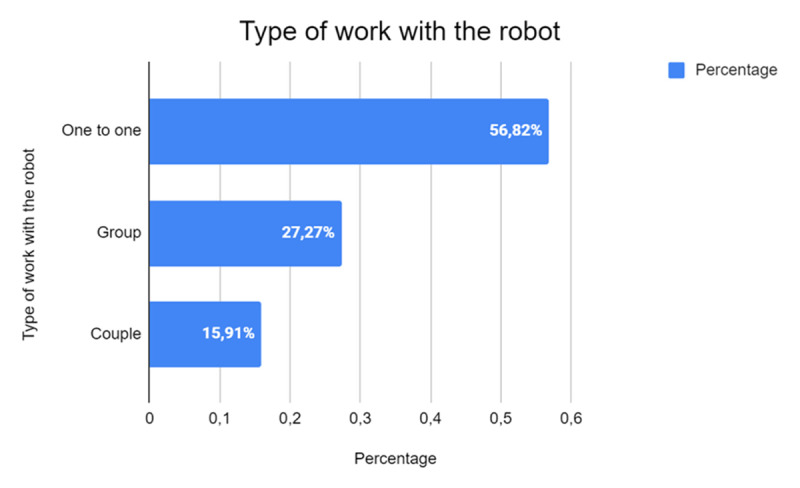
Types of Work Environments in Which Robots Are Used with Children.

The types of tasks and interactions facilitated by robots in the studies (Figure 12) reveal a predominant focus on one-to-one work, accounting for more than half of the cases (56.82%, *n* = 17). This approach highlights the centrality of direct interaction between the robot and the individual, where the robot’s ability to provide personalized feedback, guidance, or emotional support is most effectively leveraged. Such scenarios are particularly beneficial in contexts like therapy, education, or personalized learning environments, where tailored interactions can enhance engagement and outcomes.

Group settings represent the second most common use of robots, featured in 27.27% of the studies (*n* = 8). This trend reflects the growing interest in utilizing robotics to facilitate group dynamics and improve collaboration among participants. Robots in these settings often serve as mediators or co-participants, encouraging communication, coordination, and cooperative problem-solving within the group. This application underscores the potential of robotics to enrich social learning and collaborative experiences.

A smaller percentage of studies (15.91%, *n* = 5) explore the use of robots in pair work. Although less common, this mode of interaction suggests an emerging interest in understanding how robots can support small, focused work units. Robots in pair work settings may function as facilitators, providing structure and promoting interaction between the two participants.

An analysis of the types of robots used in the selected studies reveals that social robots are the most represented category, comprising 40.54% of the total (*n* = 13). This dominance underscores the increasing significance of social robotics, which is designed to enhance human interactions by fostering empathy, providing emotional support, and promoting social engagement. These robots are particularly valued in settings where interpersonal connection and relational dynamics play a significant role, such as education, therapy, or care environments.

**Figure 13:**
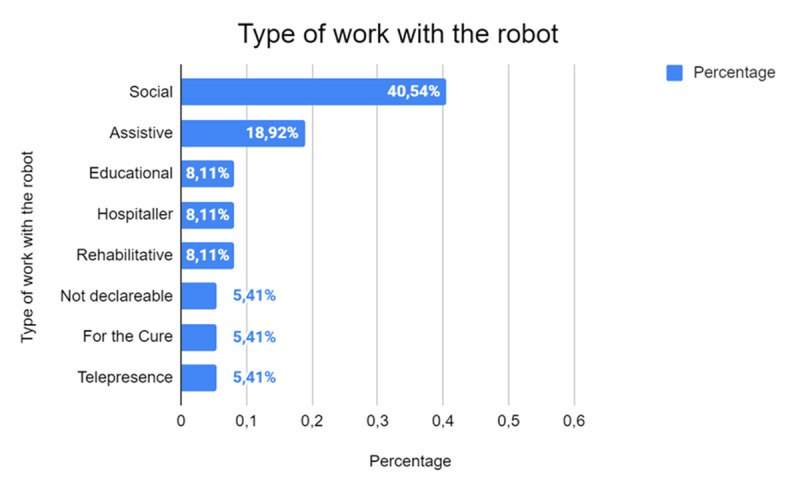
Classification of Robots Based on Their Primary Function.

As shown in Figure 13, the social function is the most used (40.54%, n = 14) indicating how there is ongoing focused research on the application of robotics in specific and impactful areas. In particular, social robots are often designed to facilitate communication, emotional support or collaborative tasks, highlighting their growing role in improving social dynamics and addressing psychological needs. These studies point to the potential of robots to improve the well-being and emotional resilience of young patients, reducing anxiety and providing companionship during difficult medical experiences.

Assistive robots follow as the second most prevalent category, accounting for 18.92% of the studies (*n* = 6). These robots are typically employed to support individuals with specific needs, such as those related to mobility, carrying out daily tasks, or cognitive assistance. Their use reflects the potential of robotics to empower individuals by enhancing independence and improving the quality of life.

Other types of robots—educational, hospital, and rehabilitation robots—are each represented in 8.11% of the studies (*n* = 3). Educational robots are utilized to facilitate learning processes, offering interactive and adaptive tools to engage students and enrich their educational experiences.

**Figure 14:**
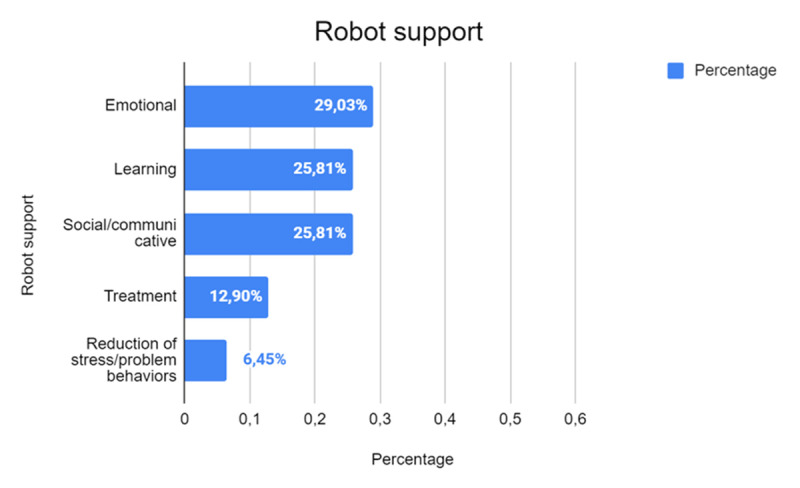
Supporting Roles of Robots in Different Contexts.

The analysis of the supporting roles assigned to robots in the studies (Figure 14) reveals a prominent focus on their emotional role, accounting for 29.03% of cases (*n* = 9). This emphasizes the psychological support that robots can provide, particularly in contexts where emotional well-being is a priority, such as healthcare, therapy, or educational settings.

Learning and sociality follow closely, each representing 25.81% of the studies (*n* = 8). These findings highlight the multifaceted applications of robots in fostering the acquisition of knowledge and enhancing social skills. In educational contexts, robots are increasingly used as interactive tools to engage learners, adapt to individual needs, and make learning more dynamic. Similarly, robots play a crucial role in promoting social interaction, helping individuals—particularly those with social or communication challenges—develop and improve their interpersonal skills through guided or facilitated activities.

Two additional roles identified are treatment (12.90%, *n* = 4) and stress reduction or the mitigation of problem behaviors (6.45%, *n* = 2). Robots used in treatment settings often assist in therapeutic interventions, rehabilitation, or medical procedures, offering support tailored to specific clinical or developmental needs. Their role in reducing stress or problem behaviors underscores their potential in managing challenging situations, creating a calming effect, or providing structured guidance to address disruptive behaviors.

**Figure 15:**
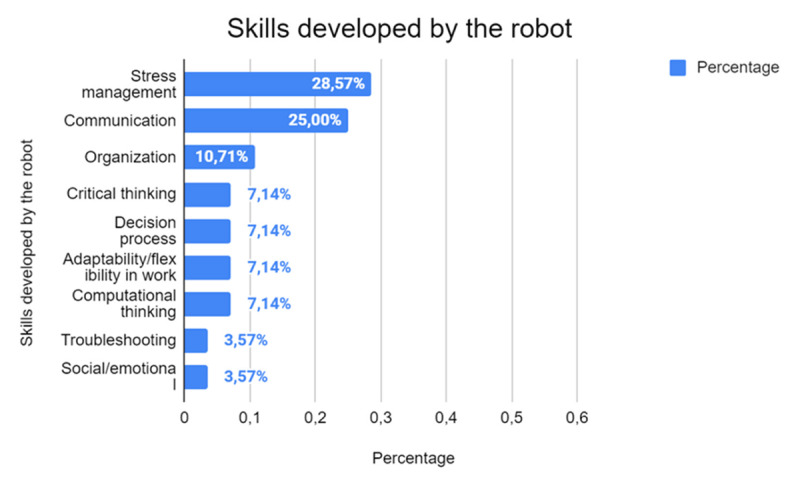
Skills Developed Through Human-Robot Interaction.

Finally (Figure 15), the analysis of skills developed using robots shows that stress management is the most frequently highlighted skill, accounting for 28.57% of studies (*n* = 8). This finding highlights the potential for robots to contribute to personal well-being, particularly in high-pressure environments such as clinical or therapeutic settings.

Communication skills follow closely at 25% (*n* = 7), reflecting the role of robots in facilitating interaction and improving verbal and non-verbal communication. This is particularly relevant in educational and therapeutic settings where developing communication skills is a key objective. Organizational skills, highlighted in 10.71% of studies (*n* = 3), demonstrate the potential of robots to assist with planning, time management and task coordination, thus promoting greater personal and group efficiency.

Cognitive skills, such as critical thinking and decision making, are less common, with less than 10% of the studies (*n* < 3). Despite this low representation, these areas hold significant potential for future research, as robots could be instrumental in challenging users to solve problems, evaluate information and make informed choices in complex scenarios. Social and emotional skills, associated with critical thinking at a lower percentage (3.57%, *n* = 1 each), highlight an emerging area where robots could help users navigate interpersonal relationships and develop emotional intelligence.

## Discussion

The findings of this systematic review confirm the growing role of robotics in hospital and home education. However, they also highlight persistent gaps in research, particularly regarding adolescent learners and long-term studies on educational impact. This discussion contextualizes these findings within the broader field of educational robotics and examines their implications for future research and practice, ensuring a direct connection between the results and the broader educational and technological landscape.

This review emphasizes the integration of educational robotics as a promising intervention to address the unique challenges of education in hospital and home settings. Robots facilitate personalized learning and reduce social isolation, ensuring continuity in the school curriculum while promoting cognitive, social, and emotional development (Belpaeme et al., 2018). Their ability to create engaging, interactive experiences helps overcome barriers that limit access to quality education for children facing long hospital stays or periods of isolation (Mubin et al., 2013).

The social and relational aspects of robots play a crucial role in hospital and home education. Robots designed for collaborative tasks encourage group participation and teamwork, while those with customizable didactic frameworks allow educators to tailor activities to individual learning needs. However, these features raise questions about accessibility, adaptability, and long-term usability in diverse contexts. Additionally, findings suggest that while these technologies show promise, their integration must be carefully designed to align with the developmental and psychological needs of students, particularly younger learners who benefit from emotional engagement and structured guidance (Ros et al., 2014).

Comparing these findings with existing literature confirms the growing interest of the scientific community in innovative approaches that combine education and technology, as noted in previous studies (Beran et al., 2015). However, unlike previous research, this review identifies an important gap: the lack of studies focused on adolescents. This group, which faces significant psychological and educational challenges, could benefit from advanced robotic technologies that integrate both technical and social skills into structured learning paths (Tanaka et al., 2007). Expanding research to include adolescents would allow a more comprehensive understanding of how robotics can support different age groups, bridging the gap between early childhood interventions and higher education applications.

Another crucial aspect is the role of design characteristics in shaping educational outcomes. While humanoid robots facilitate relational activities and social-emotional learning, programmable robots excel in fostering computational thinking and technical skills. However, current research lacks detailed insights into how these design characteristics impact different cognitive and emotional outcomes. Understanding these variables remains essential for optimizing robotic interventions in education and ensuring they cater to a broad spectrum of student needs.

Future developments in robot design should prioritize flexibility and personalization, ensuring that robots dynamically adapt to the clinical and educational needs of diverse age groups and conditions. The ability of these tools to establish empathic interactions, facilitate communication, and support psychological well-being is crucial in reducing social isolation and improving motivation to learn (van den Berghe et al., 2019).

There is also a pressing need for longitudinal studies to assess the long-term impact of educational robotics not only on academic skills but also on school reintegration and identity development. Comparative analyses between robotics-based interventions and traditional educational methodologies—such as play therapy, creative arts, or teacher-mediated learning—would offer a more holistic understanding of the pedagogical potential of robotics (Bers, 2020). Additionally, research should explore how different robot designs influence engagement, interaction complexity, and adaptability to clinical settings, providing actionable insights for educators and technologists alike.

From a practical perspective, the adoption of educational robotics in hospitals and home settings requires an interdisciplinary approach. Educators and healthcare professionals can leverage these findings to optimize their use of robotics by:

– Choosing robots based on context-specific goals: Social robots for reducing isolation and fostering social skills, programmable robots for STEM-oriented learning objectives.
– Designing flexible interventions: Adapting robot interaction modes and tasks to students’ evolving clinical and emotional conditions.
– Promoting interdisciplinary collaboration: Ensuring that educators, caregivers, and technologists work together to develop sustainable, adaptable solutions for diverse user needs.

For designers and developers of educational robotics, it is essential to enhance adaptability features, ensuring that robots can be easily reconfigured for different educational and emotional needs. This includes modular programming interfaces, customizable interaction levels, and the integration of AI-driven personalization to respond dynamically to students’ progress. Collaboration between educators, healthcare professionals, and engineers will be crucial in designing solutions that are both pedagogically effective and clinically appropriate. Future developments should prioritize affordability and ease of use to facilitate widespread adoption in hospital and home learning environments.

The results of this review confirm the educational value of robotics while opening new perspectives for inclusive and personalized learning. Robotic technologies can transform unconventional learning environments into spaces for personal and collective growth, particularly for children in fragile situations (Resnick, 2017). However, to maximize their impact, long-term research and specialized training programs for teachers and caregivers are essential for effective integration into educational practice (Pelizzari et al., 2023). By promoting equitable, inclusive, and personalized learning, robotics can help redefine the role of education in hospital and home settings, transforming challenges into opportunities for growth and empowerment (Pandey & Gelin, 2018).

The integration of robotics in hospitals and home education represents a forward-thinking pedagogical vision that goes beyond immediate challenges. With appropriate investments in research, technology, and teacher training, these tools can support a more resilient and inclusive educational ecosystem, empowering students across diverse learning environments. The findings underscore the importance of ongoing innovation in this field, highlighting the potential for robotics to bridge educational gaps and enhance the well-being of students facing unique learning conditions.

Several limitations should be acknowledged. The linguistic restriction to English and Italian may have excluded relevant studies in other languages. Additionally, by focusing only on peer-reviewed literature, grey literature and practical implementations may have been overlooked. Another limitation is the heterogeneity of studies, with variations in methodology, sample size, and objectives, making it difficult to draw generalizable conclusions. Most research also focuses on short-term effects, with limited evidence on the long-term impact of robotics in hospital and home education. Finally, despite efforts to reduce selection bias, the evolving nature of robotics means that some recent advancements may not yet be reflected in published studies. Future research should address these gaps through longitudinal studies and a broader inclusion of diverse methodologies.

While this review highlights the benefits of robotics in hospital and home education, several research gaps remain. Future studies should explore how robotics can be optimized for adolescents, a demographic often overlooked in existing research. Understanding how teenage learners engage with robotic tools—particularly in relation to autonomy, motivation, and self-regulated learning—could provide critical insights for more effective interventions.

Additionally, longitudinal studies are needed to assess the lasting impact of robotics on students’ academic performance, emotional well-being, and reintegration into traditional schooling. Research comparing robotics-based interventions with other educational approaches, such as gamification or virtual reality, could provide a more nuanced understanding of the strengths and limitations of different technologies in non-traditional learning environments.

Finally, future investigations should examine how teachers and caregivers perceive and integrate robotics into their practice. Understanding educators’ challenges in adopting robotics and identifying best practices for implementation will be key in maximizing the effectiveness of these tools. Developing frameworks for assessing the impact of robotics-based learning experiences across different medical and educational conditions could further support evidence-based integration in hospital and home settings.

## Conclusions

Integration of robotics in hospitals and home education emerges as a crucial educational strategy to ensure an inclusive and meaningful learning pathway for children forced away from mainstream schools due to prolonged illness. The SLR presented in this study highlighted how such digital tools can represent an opportunity not only to maintain continuity in schooling but also to develop fundamental skills that extend beyond mere technological literacy.

The first relevant aspect concerns the flexibility needed in the organization of education in hospital and home settings. Education in these environments must continually adapt to the changing conditions of the students, both clinically and emotionally. The personalization of teaching emerges as a central element: Each child is considered in their uniqueness, with programs and objectives that meet their specific needs. Robotics lend themselves particularly well to this dynamic, as they allow for creative, interactive approaches that can be modulated according to the individual’s abilities and progress.

Another key aspect that emerged from the review is the role of technology as a bridge between the hospital world and the school community. Through digital platforms, coding tools, and educational robots, children can continue to maintain social interactions with peers and participate in collaborative activities, reducing feelings of isolation. These technologies not only support the acquisition of cognitive skills but are also effective in enhancing emotional and social skills, key elements in fostering resilience and building a positive identity.

The diversification of technologies and robots used also requires further exploration to identify which tools are most effective in specific clinical and educational contexts. Looking forward, it is crucial to continue to explore and refine the use of innovative technologies in hospital and home education. Future developments should aim for greater customization and integration of digital technologies into the curriculum, widening access to these resources for a broader range of learners. Collaboration between educators, health professionals, and technology developers is essential for creating increasingly effective solutions capable of meeting the complex challenges of these contexts.

Robotics represents a forward-looking pedagogical tool that has the potential to redefine education in hospitals and home settings. By addressing cognitive, social, and emotional needs, robots empower students to overcome challenges associated with prolonged illness, fostering resilience and creating new opportunities for growth. However, realizing this potential requires continued investment in research and training, as well as the development of adaptable and inclusive technologies. As educators and practitioners embrace these innovations, the future of hospital and home education hold promises for more equitable, personalized, and impactful learning experiences.

## Additional Files

The additional files for this article can be found as follows:

10.5334/cie.156.s1 Supplementary File 1. Studies included in the SLR.

10.5334/cie.156.s2 Supplementary File 2. Dataset of analysis for the SLR.
