# Supplementary material for: Integrating Robotics in Hospital and Home Education: A Systematic Review of Innovative Teaching Practices
Source: Contin Educ. 2025 Sep 1;6(1):135–61. doi: 10.5334/cie.156 (PMC12412443; doi:10.5334/cie.156)
Supplement: Supplementary File 1. — Studies included in the SLR. [file cie-6-1-156-s1.pdf]

Integrating Robotics in Hospital and Home Education. A Systematic Review of Innovative Teaching Practices

Supplementary Material 1

Pelizzari Federica, Rocco Simone, Ferrari Simona

Università Cattolica del Sacro Cuore (Italy)

Studies Included in the SLR

Number	***Authors***	***Title***	***Publication Year***
**1**	Connolly C. et al.	Exploring new frontiers of education using humanoid robots–a case study of patient centred innovation in digital health education	2022
**2**	Alemi M. et al.	Effect of utilizing a humanoid robot as a therapy-assistant in reducing anger, anxiety, and depression	2014
**3**	Meghdari A. et al.	Arash: A social robot buddy to support children with cancer in a hospital environment	2018
**4**	González-González CS. Et al.	Computational thinking and down syndrome: An exploratory study using the KIBO robot	2019
**5**	Ehsan H. et al.	Examining the role of parents in promoting computational thinking in children: A case study on one homeschools family (fundamental)	2019
**6**	Kim Y, Tscholl M	Young children's embodied interactions with a social robot	2021
**7**	Stiti S. et al.	Innovative protocol of an exploratory study evaluating the acceptability of a humanoid robot at home of deaf children with cochlear implants	2023
**8**	Robinson NL. et al.	A Social Robot to Deliver an 8-Week Intervention for Diabetes Management: Initial Test of Feasibility in a Hospital Clinic	2020
**9**	Lavigne HJ. et al.	Computational thinking with families: Studying an at-home media intervention to promote joint media engagement between preschoolers and their parents	2023
**10**	Soares N, Kay JC, Craven G	Mobile Robotic Telepresence Solutions for the Education of Hospitalized Children	2017
**11**	Newhart VA, Olson JS	My student is a robot: How schools manage telepresence experiences for students	2017
**12**	Littler BK. et al.	Reducing negative emotions in children using social robots	2021

The original article can be downloaded from https://doi.org/10.5334/cie.NNN

**13**	Jeong S. et al.	A Social Robot to Mitigate Stress, Anxiety, and Pain in Hospital Pediatric Care	2015
**14**	Logan DE. et al.	Social robots for hospitalized children	2019
**15**	Schmucker M. et al.	Mobile robotic telepresence between hospital and school: Lessons learned	2020
**16**	Worlikar H. et al.	Is it feasible to use a humanoid robot to promote hand hygiene adherence in a hospital setting?	2021
**17**	González-González CS, Violant-Holz V, Gil-Iranzo RM	Social Robots in Hospitals	2021
**18**	Pourteimour S, Kazemi S	The effectiveness of the robotic game kit on anxiety among hospitalized preschool children: A non-randomized controlled trial	2021
**19**	Ramachandran BR, Lim JC	User validation study of a social robot for use in hospital wards	2021
**20**	Chang CY. et al.	Effects of robot-assisted digital storytelling on hospitalized children's communication during the COVID-19 pandemic	2023
**21**	Lytridis C. et al.	Distance special education delivery by social robots	2020
**22**	Giannopulu I, Pradel G	From child-robot interaction to child-robot-therapist interaction: A case study in autism	2012
**23**	Alemi M. et al.	Impact of a social humanoid robot as a therapy assistant in children cancer treatment	2014
**24**	Baroni I. et al.	What a robotic companion could do for a diabetic child	2014
**25**	Tanaka F. et al.	Pepper learns together with children: Development of an educational application	2015
**26**	Henkemans OA. et al.	Design and evaluation of a personal robot playing a self-management education game with children with diabetes type 1	2017
**27**	Michaelis JE, Mutlu B	Someone to read with: Design of and experiences with an in-home learning companion robot for reading	2017
**28**	Scassellati B. et al.	Improving social skills in children with ASD using a long-term, in-home social robot	2018
**29**	Yamamoto R, Sekimoto H, Kubota K	Studying a Tele-Presence Robot Installed into a Hospital Classroom	2016
**30**	Meghdari A. et al.	Design Performance Characteristics of a Social Robot Companion Arash for Pediatric Hospitals	2018
**31**	Kyrarini M. et al.	A Survey of Robots in Healthcare	2021
