# Supplementary material for: Integrating Robotics in Hospital and Home Education: A Systematic Review of Innovative Teaching Practices
Source: Contin Educ. 2025 Sep 1;6(1):135–61. doi: 10.5334/cie.156 (PMC12412443; doi:10.5334/cie.156)
Supplement: Supplementary File 2. — Dataset of analysis for the SLR. [file cie-6-1-156-s2.pdf]

# Integrating Robotics in Hospital and Home Education. A Systematic Review of Innovative Teaching Practices

*Supplementary Material 2*

**Pelizzari Federica, Rocco Simone, Ferrari Simona**

*Università Cattolica del Sacro Cuore (Italy)*

Supplementary Table 2

Summary of the dataset analysis conducted for the Systematic Literature Review (SLR). The coding scheme applied is detailed in the accompanying notes.

| Citation APA | Type of disorder/ illness | Setting of use | Recipients of the intervention | type of robot | Appearance of the robot | Type of work with the robot | Interaction with the robot | Didactic/ educational purpose | skills developed by the robot: | Robot support | Mode of use | Objectives/research questions | Research methods | Framework cited | type of study |
|---|---|---|---|---|---|---|---|---|---|---|---|---|---|---|---|
| Giannopulu I et al., 2012 | 1 | 6 | 1 | 1 | 3 | 1 | 3 | 8 | 13 | 3 | 1 | 2 | 7 | 0 | 4 |
| Alemi M et al., 2014 | 3 | 1 | 1 | 3 | 1 | 2 | 4 | 6 | 9 | 4 | 1 | 3 | 8 | 10 | 3 |
| Alemi M et al., 2014 | 3 | 1 | 1 | 3 | 1 | 2 | 3 | 8 | 8 | 4 | 1 | 2 | 1 | 10 | 3 |
| Baroni I et al., 2014 | 4 | 1 | 1, 2 | 1 | 1 | 1 | 3, 4 | 8 | 8 | 3 | 1 | 1 | 1, 7 | 10 | 1 |
| Jeong S et al., 2015 | 10 | 1 | 1 | 1 | 2 | 3 | 3 | 6 | 8 | 4 | 1 | 2 | 1,8 | 7 | 3 |
| Tanaka F et al., 2015 | 0 | 4 | 1 | 2 | 1 | 1, 3 | 3, 4 | 1 | 0 | 1 | 6 | 4 | 7 | 8, 9 | 5 |

| | | | | | | | | | | | | | | | |
|---|---|---|---|---|---|---|---|---|---|---|---|---|---|---|---|
| Yamamoto R et al., 2016 | 12 | 5 | 1 | 7 | 4 | 1 | 5 | 1 | 6 | 1 | 6 | 4 | 7 | 6 | 5 |
| Soares N et al., 2017 | 10 | 5 | 1 | 3 | 3 | 1 | 4 | 7 | 1 | 3 | 7 | 5 | 1,7 | 6 | 2 |
| Newhart VA et al., 2017 | 10 | 5 | 1 | 3 | 1 | 1 | 4 | 7 | 1 | 3 | 7 | 5 | 3 | 6 | 1 |
| Henkemans OA et al., 2017 | 4 | 1 | 1 | 1 | 1 | 1 | 3, 4 | 5 | 7 | 1 | 2 | 3 | 6, 8 | 0 | 3 |
| Michaelis JE et al., 2017 | 0 | 2 | 1 | 1 | 1 | 1 | 3, 4 | 5 | 0 | 3 | 3 | 2 | 1, 6 | 0 | 5 |
| Meghdari A et al., 2018 | 3 | 1 | 1 | 1 | 1 | 0 | 3 | 5 | 8 | 4 | 1 | 1 | 8 | 7 | 3 |
| Meghdari A et al., 2018 | 3 | 1 | 1 | 1 | 1 | 1,2 | 1 | 8 | 5 | 3 | 4 | 1 | 1,3,8 | 10 | 3 |
| Scassellati B et al., 2018 | 1 | 2 | 1 | 1 | 3 | 1 | 2, 4 | 7 | 1 | 3 | 1 | 5 | 1, 5 | 7 | 5 |
| González-González CS et al., 2019 | 5 | 8 | 1,3 | 1 | 3 | 1 | 2 | 1 | 12 | 1 | 3 | 4 | 3,5,7 | 1 | 1 |
| Ehsan H et al., 2019 | 0 | 2 | 5 | 0 | 0 | 0 | 0 | 0 | 12 | 0 | 0 | 0 | 5,7 | 2 | 4 |
| Logan DE et al., 2019 | 10 | 1 | 1 | 1 | 2 | 1 | 3 | 6 | 8 | 4 | 1 | 2 | 1,5 | 7 | 5 |

| | | | | | | | | | | | | | | | |
|---|---|---|---|---|---|---|---|---|---|---|---|---|---|---|---|
| Robinson NL et al., 2020 | 4 | 1 | 3 | 1 | 1 | 2 | 4 | 5 | 6 | 1 | 2 | 3 | 8 | 0 | 2 |
| Schmucker M et al., 2020 | 10 | 5 | 1 | 7 | 0 | 0 | 5 | 7 | 0 | 3 | 7 | 5 | 3,4 | 6 | 4 |
| Lytridis C et al., 2020 | 1 | 2 | 1 | 1 | 1 | 1 | 5 | 1 | 0 | 1 | 6 | 5 | 3 | 0 | 3 |
| Kyrarini M et al., 2021 | 11 | 1,2 | 1, 4 | 3, 4, 5, 6 | 1,3,4, | 1, 2, 3, | 1,2,3,4 | 1 | 1,7,9, | 2 | 1,5 | 2,3 | 0 | 13 | 6 |
| Kim Y et al., 2021 | 0 | 2 | 1 | 2 | 1 | 1,2 | 3 | 1,7 | 1,3 | 1 | 3,6 | 2 | 4,7 | 12 | 5 |
| Littler BK et al., 2021 | 10 | 1 | 1 | 3 | 1,2 | 1 | 3 | 5 | 8 | 4 | 1 | 2 | 4 | 7 | 6 |
| Worlikar H et al., 2021 | 0 | 1 | 2 | 2 | 1 | 1 | 4 | 7 | 5 | 1 | 2 | 0 | 7 | 7 | 2 |
| González-González CS et al., 2021 | 10 | 1 | 1,2,3,4, | 1,3,5,6 | 1,2 | 1 | 3 | 5 | 8 | 4 | 1 | 2 | 4 | 7 | 6 |
| Pourteimour S et al., 2021 | 10 | 1 | 1 | 1 | 3 | 2 | 3 | 6 | 8 | 4 | 1 | 3 | 2,5 | 10 | 3 |
| Ramachandran BR et al., 2021 | 0 | 1 | 2 | 5 | 1 | 1 | 5 | 7 | 0 | 2 | 0 | 3 | 1,3 | 7 | 1 |
| Connolly C et al., 2022 | 4 | 7 | 2 | 4 | 1 | 1 | 4 | 1 | 7 | 2 | 2 | 3 | 1,2,5,8 | 3 | 2 |

This document contains supplementary material for the above-mentioned article, as provided by the authors.

The original article can be downloaded from https://doi.org/10.5334/cie.156

| | | | | | | | | | | | | | | | |
|---|---|---|---|---|---|---|---|---|---|---|---|---|---|---|---|
| Stiti S et al., 2023 | 8 | 1 | 1,5 | 6 | 1 | 1 | 3 | 7 | 1 | 2 | 1 | 3 | 1,8 | 7 | 4 |
| Lavigne HJ et al., 2023 | 0 | 2 | 1,5 | 0 | 0 | 0 | 0 | 0 | 0 | 0 | 0 | 0 | 3 | 2 | 3 |
| Chang CY et al., 2023 | 7 | 1 | 1 | 1 | 3 | 2 | 1 | 8 | 1 | 4 | 4 | 2 | 1 | 10 | 3 |

Note. The following coding scheme was applied across all table variables:

**Type of disorder/illness (1–12):**

1 = Autism
2 = Leukemia
3 = Cancer
4 = Diabetes
5 = Down syndrome
6 = Food-related disorders
7 = Anxiety, fear, depression, anger
8 = Visual/auditory impairments
9 = Mental disorders
10 = Pediatric diseases
11 = Disability
12 = Viral infections

**Setting of use (1–8):**

1 = Hospital
2 = Home
3 = In-hospital school
4 = Home education
5 = Telepresence
6 = Day hospital
7 = Clinic
8 = Association

**Recipients of the intervention (1–6):**

1 = Children (0–12 years)
2 = Adults (20–70 years)
3 = Adolescents (13–19 years)
4 = Elderly (≥71 years)
5 = Parents/families
6 = Teachers/educators

**Type of robot (1–7):**

1 = Social
2 = Educational
3 = Assistive
4 = Care-focused
5 = Hospital service
6 = Rehabilitation
7 = Telepresence

**Appearance of the robot (1–5):**

1 = Humanoid
2 = Animal-like
3 = Technological
4 = Tablet-based
5 = Smartphone-based

**Type of work with the robot (1–3):**

1 = One-to-one
2 = Group
3 = Pair

**Mode of interaction (1–5):**

1 = Proximal facial expression
2 = Proximal manual
3 = Proximal bodily
4 = Proximal vocal
5 = Remote

**Didactic/educational purpose (1–9):**

1 = With the robot
2 = To the robot
3 = To educate the robot
4 = For the robot
5 = Motivator
6 = Distractor

7 = Facilitator
8 = Entertainer
9 = Tutor

**Robot support function (1–5):**

1 = Learning
2 = Caring
3 = Social/communicative
4 = Emotional
5 = Attentive

**Mode of use (1–6):**

1 = Social, relational, affective/empathic
2 = Self-regulatory and metacognitive
3 = Motivational (involvement, interest, participation)
4 = Expressive/narrative, creative and divergent thinking
5 = Practical-motor
6 = Cognitive/literacy

**Objectives/research questions (1–5):**

1 = Robot functionality
2 = Person–robot interaction
3 = Disease management via robot
4 = Educational application of robot
5 = Communication through robot

**Research methods (1–8):**

1 = Questionnaire
2 = Pre-/post- and/or follow-up psychological tests
3 = Interviews/discussions/focus groups
4 = Grounded theory
5 = Video/audio documentation
6 = Video analysis
7 = Field observations
8 = Controlled experiments with robot evaluation

**Framework cited (1–13):**

1 = Coding/programming
2 = Computational thinking
3 = Problem solving
4 = STEM
5 = CCPS

6 = Mobile robotic telepresence (MRT)
7 = Social robotics
8 = CRR (care-receiving robot)
9 = TPR (total physical response)
10 = Hospitalization, robots and stress reduction
11 = HRI (human–robot interaction)
12 = Embodied cognition
13 = Artificial intelligence (AI)

**Type of study (1–6):**

1 = Exploratory
2 = Feasibility
3 = Experimental (control vs. experimental groups)
4 = Case study (single/pilot)
5 = Multiple complementary or parallel case studies
6 = Literature review

This document contains supplementary material for the above-mentioned article, as provided by the authors.

The original article can be downloaded from https://doi.org/10.5334/cie.156
